# Identifying Different Immunoresistance Risk Profiles Among Experienced Aesthetic Botulinum Neurotoxin A Recipients: A Latent Class Analysis

**DOI:** 10.1111/jocd.16686

**Published:** 2024-12-08

**Authors:** Fang‐Wen Tseng, Vasanop Vachiramon, Michael H. Gold, Tatjana Pavicic, Clifton M. Tay, Gerard W. Toh, Diana M. K. Tan, Je‐Young Park

**Affiliations:** ^1^ Everbeaute Medical Aesthetics Taipei Taiwan; ^2^ Division of Dermatology, Faculty of Medicine, Ramathibodi Hospital Mahidol University Bangkok Thailand; ^3^ Gold Skin Care Center Nashville Tennessee USA; ^4^ Tennessee Clinical Research Center Nashville Tennessee USA; ^5^ Private Practice for Dermatology & Aesthetics Dr. Tatjana Pavicic Munich Germany; ^6^ Merz Aesthetics Asia Pacific Singapore Singapore; ^7^ Tech Observer Asia Pacific Singapore Singapore; ^8^ Advocates for Advancing Health Singapore Singapore; ^9^ Apkoo‐Jung Oracle Dermatology Clinic Seoul Korea

**Keywords:** botulinum toxin A, consumer behavior, health knowledge, attitudes, practice, immunoresistance, injectables, neuromodulator, secondary nonresponse

## Abstract

**Background:**

Immunoresistance to botulinum neurotoxin A (BoNT‐A) due to neutralizing antibodies (NAbs) can lead to partial or complete secondary nonresponse (SNR), potentially limiting individuals' aesthetic and/or medical therapeutic options in the short and/or long term. Understanding factors directly or indirectly influencing BoNT‐A immunoresistance risk is crucial.

**Aims:**

This analysis explored patterns of latent risk factors (biological and behavioral) that may influence the risk of developing BoNT‐A immunoresistance among experienced aesthetic BoNT‐A recipients.

**Methods:**

Latent class analysis (LCA) was applied to survey data from 363 experienced BoNT‐A recipients from six Asia‐Pacific countries to identify distinct subgroups based on their patterns of risk factor or risk proxy variables. The five risk proxy variables used for modeling capture information on BoNT‐A treatments (treatment indications/locations as proxies for dose), symptoms of declining efficacy, number of aesthetic treatments over the past 3 years, and clinic and BoNT‐A formulation switching behaviors. These represent established risk factors and treatment‐seeking behaviors suggested to influence immunoresistance risk.

**Results:**

LCA identified 3 distinct profiles of individuals, which we described based on the observed patterns of risk proxies as: “lower‐risk” (55%), “moderate‐risk” (39%), and “higher‐risk” (6%). Individuals in the “higher‐risk” profile reported higher BoNT‐A exposure, more symptoms of declining efficacy, and distinct patterns of knowledge and attitudes toward BoNT‐A immunoresistance that could account for their treatment‐seeking behaviors.

**Conclusions:**

This study suggests that individual behaviors (the “human factor”) have a notable influence on BoNT‐A immunoresistance risk. Gaining deeper insights into these factors could support more targeted and effective interventions to mitigate risk.

## 1 Introduction

Botulinum neurotoxin A (BoNT‐A) injections are the leading nonsurgical aesthetic procedure globally across all genders and age groups [1]. With steadily increasing trends of aesthetic BoNT‐A use, especially for long‐term treatment at regular intervals, the potential for immunoresistance development after repeated treatments due to the formation of BoNT‐A neutralizing antibodies (NAbs) is a significant concern. NAbs can contribute to partial or complete secondary nonresponse (SNR) to BoNT‐A neuromodulation, which may limit individuals' therapeutic options [2, 3, 4]. These implications are relevant for healthcare professionals (HCPs) in different disciplines (e.g., neurology and aesthetic medicine), and for patients who benefit from aesthetic and/or medical BoNT‐A treatment.

Consequently, it has been proposed that approaches compatible with continued or future BoNT‐A treatment should focus on achieving responsiveness to therapy while limiting the stimulation of the host immune response [2, 3, 4]. This may involve the use of any/all of: lower immunogenicity BoNT‐A formulations, the lowest effective dose, and the longest treatment interval acceptable to patients. With these guiding principles, the multidisciplinary AeSthetic Council on Ethical use of Neurotoxin Delivery (ASCEND) has worked to encourage informed discourse on BoNT‐A immunoresistance and advocate for sustainable use of BoNT‐A in aesthetic practice [4, 5]. Given the paucity of data on BoNT‐A immunoresistance in real‐world practice, ASCEND advocated for research to inform shared decision‐making with aesthetic patients [4]. A consumer survey was conducted in six Asia‐Pacific countries to explore the implications of BoNT‐A immunoresistance from the aesthetic patient's perspective [5]. Even among experienced aesthetic BoNT‐A recipients, many of whom reported multiple symptoms suggestive of declining BoNT‐A efficacy, the level of understanding of immunoresistance and its associated risk factors was surprisingly limited [5].

**FIGURE 1:**
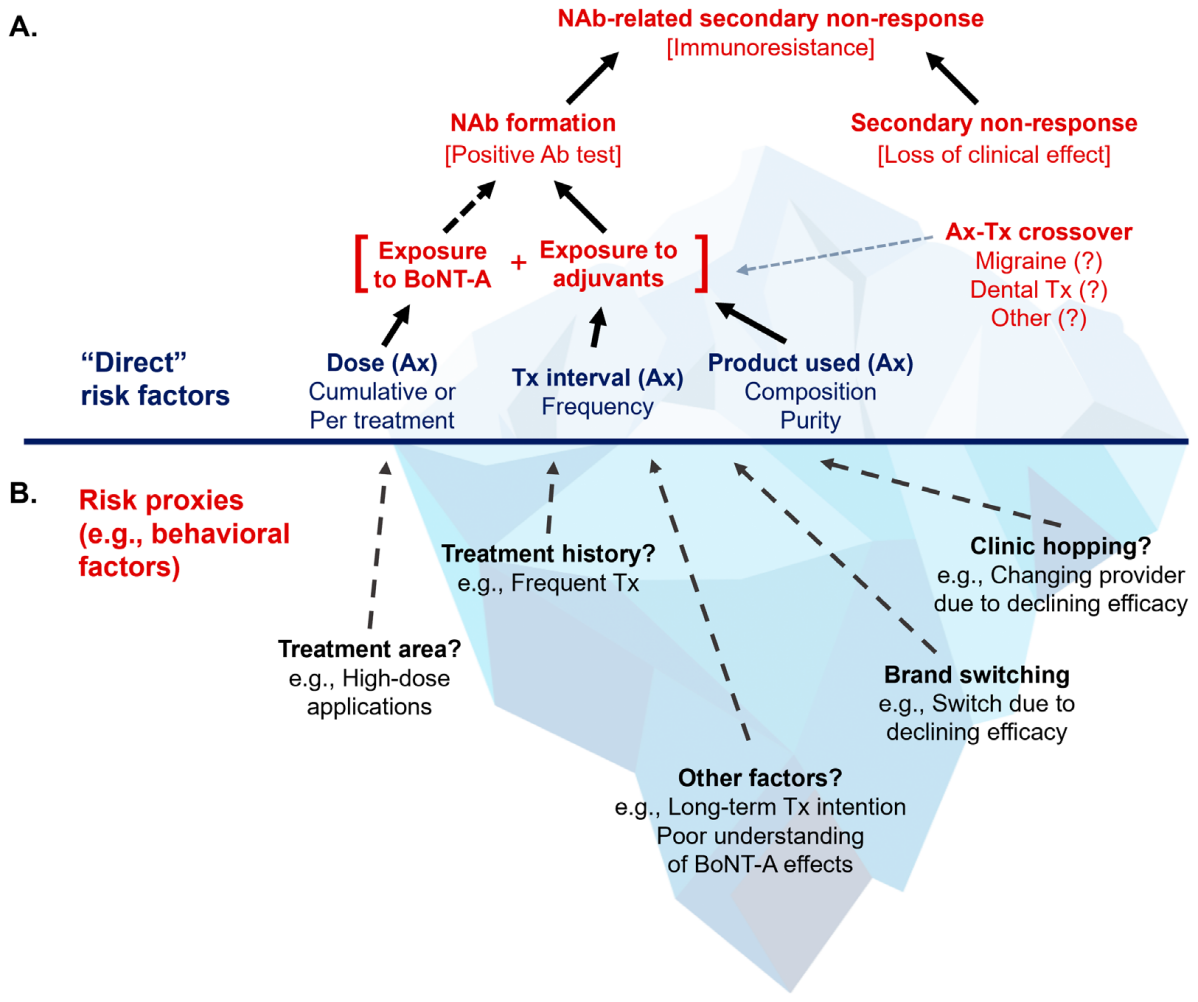
Schematic showing factors that can potentially influence the risk of developing BoNT‐A immunoresistance either (A) directly or (B) indirectly. Ax, aesthetic treatments; NAb, neutralizing antibodies; SNR, secondary non‐response; Tx, treatment.

Factors that directly influence the risk of BoNT‐A immunoresistance have been described in the literature. These include the purity and immunogenicity of BoNT‐A formulation used, the doses administered, and treatment interval or frequency (Figure 1A) [2, 3, 4, 6]. These risk factors are well described biologically and mechanistically but represent merely the “tip of the iceberg” in understanding immunoresistance development and risk in real‐world aesthetic practice. Controlled studies poorly recapitulate real‐life aesthetic practice settings, as they are designed to demonstrate efficacy and focus on specific indicators over a limited timeframe with minimal consideration of NAb‐related SNR, which generally occurs after repeated treatments over an extended period. On the other hand, longitudinal clinical studies on immunoresistance development are largely infeasible due to the necessity of following up large patient cohorts for an extended period of time. Additionally, access to mouse hemidiaphragm assay [MHDA, [7]], which is the gold standard test for NAbs, is a challenge in routine clinical practice. There is also the difficulty of translating “average” risk inferred from studies to the risk for an individual one sees in the clinic.

We believe that part of the solution lies in addressing the “human factor,” including knowledge, attitudes and treatment‐seeking behaviors, that influence an individual's risk of BoNT‐A immunoresistance (Figure 1B). This is an aspect that remains very much underexplored in the literature. Since the known risk factors (e.g., BoNT‐A doses, immunogenicity of formulations, and presence of NAbs) are challenging to quantify precisely in real‐world studies, we explored the use of risk‐related characteristics (proxies of risk) to identify distinct profiles of individuals with differing risk levels within a real‐world survey dataset. To accomplish this, we utilized the latent class analysis [LCA; [8, 9]] technique together with potential risk proxy variables in the dataset (e.g., reported treatment history, exposure to BoNT‐A, and use of multiple BoNT‐A formulations/brands) (Figure 1B) to identify subgroups of survey respondents with different levels of potential immunoresistance risk. Our analysis complements existing perspectives on risk derived from analysis of clinical studies and could offer actionable insights for both aesthetic patients and healthcare practitioners.

## 2 Methods

### 2.1 Dataset Characteristics

This analysis utilized data from a survey of experienced BoNT‐A recipients in six Asia‐Pacific countries/territories. The survey was designed to study the typical treatment journey of experienced aesthetic BoNT‐A recipients in the APAC region while also assessing their awareness and attitudes relating to BoNT‐A immunoresistance and its impact on treatment effectiveness. Respondents (*n* = 363, recruited using online consumer panels in Australia, Hong Kong SAR China, Singapore, South Korea, Taiwan, and Thailand) had undergone ≥ 6 BoNT‐A treatments for facial and/or body indications within the past 36 months. All participants were female, aged 21–55, which reflects the demographic trend that most aesthetic BoNT‐A recipients are female [1]. They reported having a middle to high monthly household income and expressed intentions for future BoNT‐A treatments.

### 2.2 Analysis Methods

This analysis was an exploratory investigation to determine whether the individuals participating in the survey could be categorized into distinct subgroups based on their risk proxy profiles. We also asked how these profiles are related to the level of risk/potential for developing BoNT‐A immunoresistance.

#### 2.2.1 Variables and Selection of Risk Proxies

**FIGURE 2:**
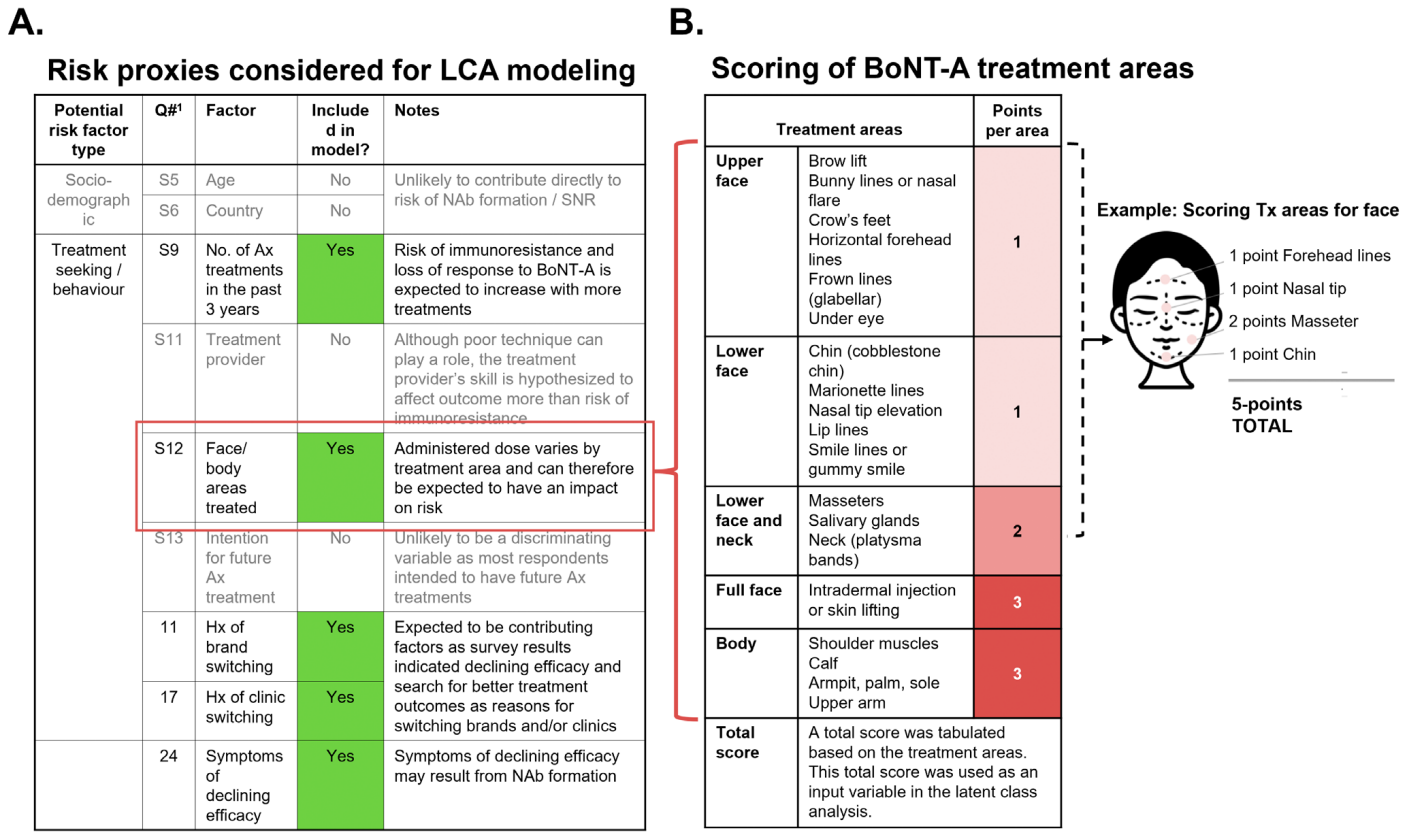
(A) Risk proxies considered for LCA modeling. (B) Scoring of BoNT‐A treatment areas used as a proxy for dosage. Abbreviations: Ax, aesthetic treatments; NAb, neutralizing antibodies; Hx, historyS5: How old were you on your last birthday? S6: Which city do you live in? S9: How many times did you have Ax treatment(s) with botulinum toxin in the past 3 years. S11: Who administered your most recent Ax treatment with botulinum toxin? S12: Which of these areas have you ever had Ax treatment(s) with botulinum toxin performed? S13: Are you planning to have one or more Ax treatments on any parts of the face and/or body (including touch‐ups) in the next 6 months? Q11: How many times did you switch brand(s)? Q17: In your aesthetic journey experience, have you switched clinic(s) from the time you first underwent Ax treatments with botulinum toxin? Q24: Did you experience any of these symptoms after undergoing Ax treatments with botulinum toxin?

For modeling, measures representing potential risk concepts/factors or risk proxies were constructed using the available survey variables (Figure 2A). We targeted risk concepts known to be linked to BoNT‐A immunoresistance/NAb‐related SNR from earlier studies (e.g., dose, treatment interval, product composition, and/or purity [Figure 1]) [2, 3, 4, 6]. The proxy measures for BoNT‐A exposure included in the study were the > 8 treatments received by patients over the past 3 years as the main proxy for treatment interval and areas of the face/body treated as the main proxy for dose. We used treated area as a proxy for dose by utilizing a scoring system that categorizes the treatment areas into upper face (scoring 1 point each), lower face/neck (with two subsections scoring 1–2 points each), full face (scoring 3 points), and body (scoring 3 points each). As such, the points assigned to each area reflect the BoNT‐A dose commonly administered in clinical practice. Based on this, a patient treated in the horizontal forehead lines (1 point), nasal tip (1 point), masseters (2 points), and chin (1 point) would accumulate a total score of 5 points (Figure 2B). In addition, we also included measures of symptoms that could indicate declining BoNT‐A efficacy and measures related to treatment‐seeking behaviors, such as respondents' reported history of clinic switching and brand switching, which has been associated with a higher rate of NAb formation [10] (Figure 2A).

#### 2.2.2 LCA Modeling and Profiles of Latent Risk Classes

We aimed to discover whether subgroups characterized by different levels of risk exist within our survey sample. To achieve this, we conducted preliminary analyses using the LCA, k‐means clustering, and hierarchical clustering techniques. We selected LCA as our preferred method, as it produced the most stable and meaningful groupings within the sample.

Latent class analysis is a statistical technique to discover subgroups, or “latent classes,” within a population that are qualitatively distinct yet not readily identifiable. It has been applied in both social sciences and healthcare studies to identify meaningful patterns in individual behaviors and health risks/exposures within populations [11, 12]. LCA is a person‐oriented approach where each latent class is identified based on individuals' characteristics, such as similar response patterns across survey questions or scales. Each latent class represents a group of respondents that are similar to one another yet distinguishable from other groups within the population. LCA is able to use a range of data types (e.g., categorical and numerical) and allows for quantitative evaluation of model fit parameters to assess the appropriateness of the model.

**FIGURE 3:**
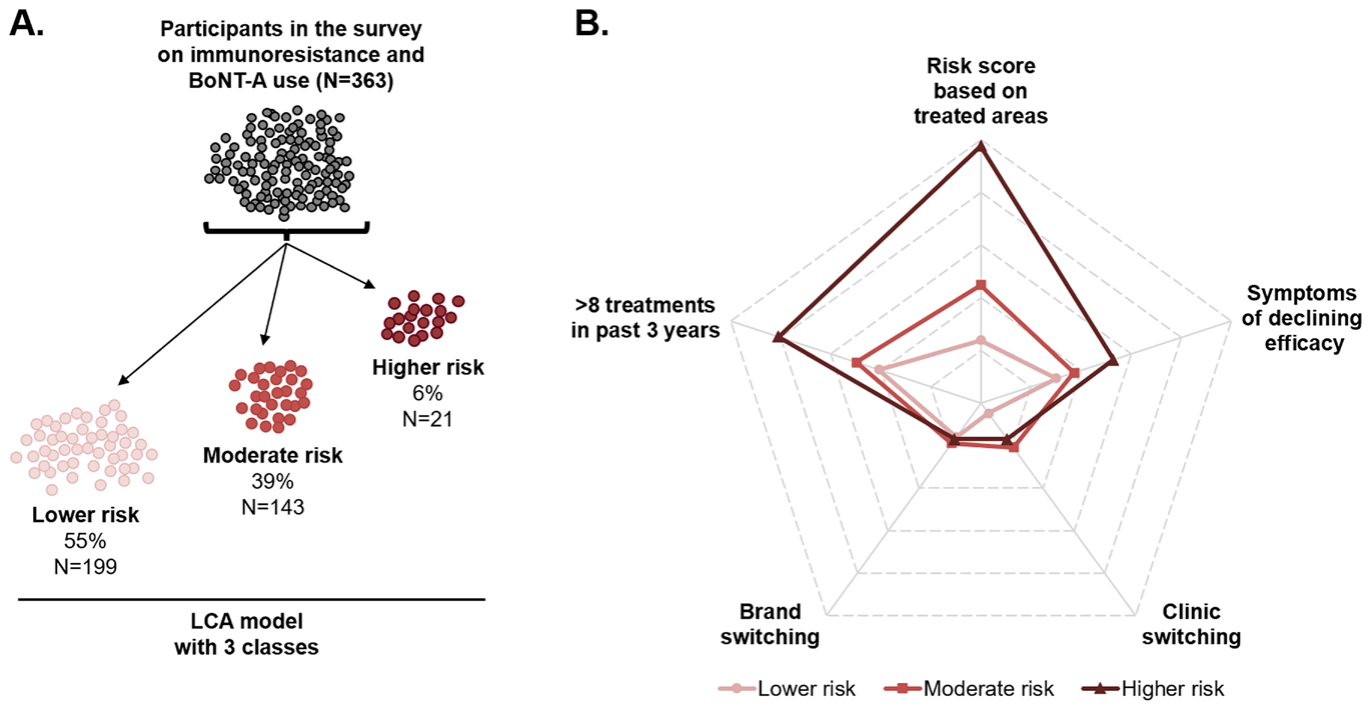
(A) Three latent risk profiles were identified using LCA and described as “lower‐risk,” “moderate‐risk,” and “higher‐risk” based on the (B) observed patterns across five risk proxy variables.

Using the five risk proxy variables, a series of latent class models (2, 3, or 4 classes) was fitted using the depmixS4 R package [13]. To identify the optimal model (i.e., the one with the number of classes that best reflected the unique response patterns within the sample), the models were compared based on model fit metrics. The appropriate model was selected based on considerations of: (1) statistical model fit parameters (i.e., likelihood ratio test [LRT], Akaike information criterion [AIC], and Bayesian information criterion [BIC]) and (2) differentiation of profile patterns across the five variables in the LCA model (Figure 3A).

The latent classes in the best‐fit 3‐class model were subsequently profiled in terms of risk proxy variables, sociodemographic, attitudinal, knowledge, and behavioral variables to describe the patterns characterizing each group.

## 3 Results

### 3.1 Three Latent Risk Profiles Identified Using LCA

The LCA modeling results indicated three distinct profiles based on the risk proxy variables, which we describe as “lower‐risk” (*n* = 199; 55%), “moderate‐risk” (*n* = 143; 39%), and “higher‐risk” (*n* = 21; 6%), as shown in Figure 3A.

The 3‐class model was chosen as the most parsimonious model, that is, the lowest‐complexity model with a statistically good fit to the data, among those tested (Table S1). Both LRT and AIC indices generally favor more complex models, whereas BIC offers a good balance between over‐fitting and generalizability and is recommended for most analyses [9]. Since both 3‐class and 4‐class models showed reasonable goodness‐of‐fit, the 3‐class model, which had the lowest BIC, was selected. It was also considered that the 3‐class model would support clearer messaging and targeted strategies for each group, thereby enhancing the practical applicability of the findings to a clinical setting.

The plot in Figure 3B demonstrates how the three risk profiles differed in terms of the five risk proxy variables. Individuals in the “higher‐risk” profile had higher BoNT‐A treatment area scores on average than those in the “moderate‐risk” and “lower‐risk” profiles (Figure 3B). This pattern indicates greater exposure to BoNT‐A (number of areas treated and/or high‐dose applications) and thus potentially increased risk of developing BoNT‐A resistance.

Similarly, the mean number of symptoms of declining efficacy and the number of aesthetic treatments received over the past 3 years were also higher for participants in the “higher‐risk” profile (Figure 3B). The mean number of clinic switching was higher among those in both “moderate‐risk” and “higher‐risk” profiles compared with the “lower‐risk” group. For brand switching, there was less differentiation between the “moderate‐risk” and “higher‐risk” profiles, although both were still higher than the “lower‐risk” group (Figure 3B).

### 3.2 Knowledge and Attitudinal Differences Between the Three Latent Risk Profiles

Next, we explored how these groups differed in terms of other variables that are not known to be associated with BoNT‐A immunoresistance risk and are not readily identifiable as proxies for known risk factors (Figure 1). These included participants' sociodemographic characteristics and attitudinal and knowledge characteristics that may influence their treatment‐seeking behavior. While these characteristics may not appear to contribute directly to BoNT‐A immunoresistance risk, understanding their patterns within the identified risk groups may provide valuable context and insights into the real‐world “human” and “behavioral” factors contributing to this risk.

**FIGURE 4:**
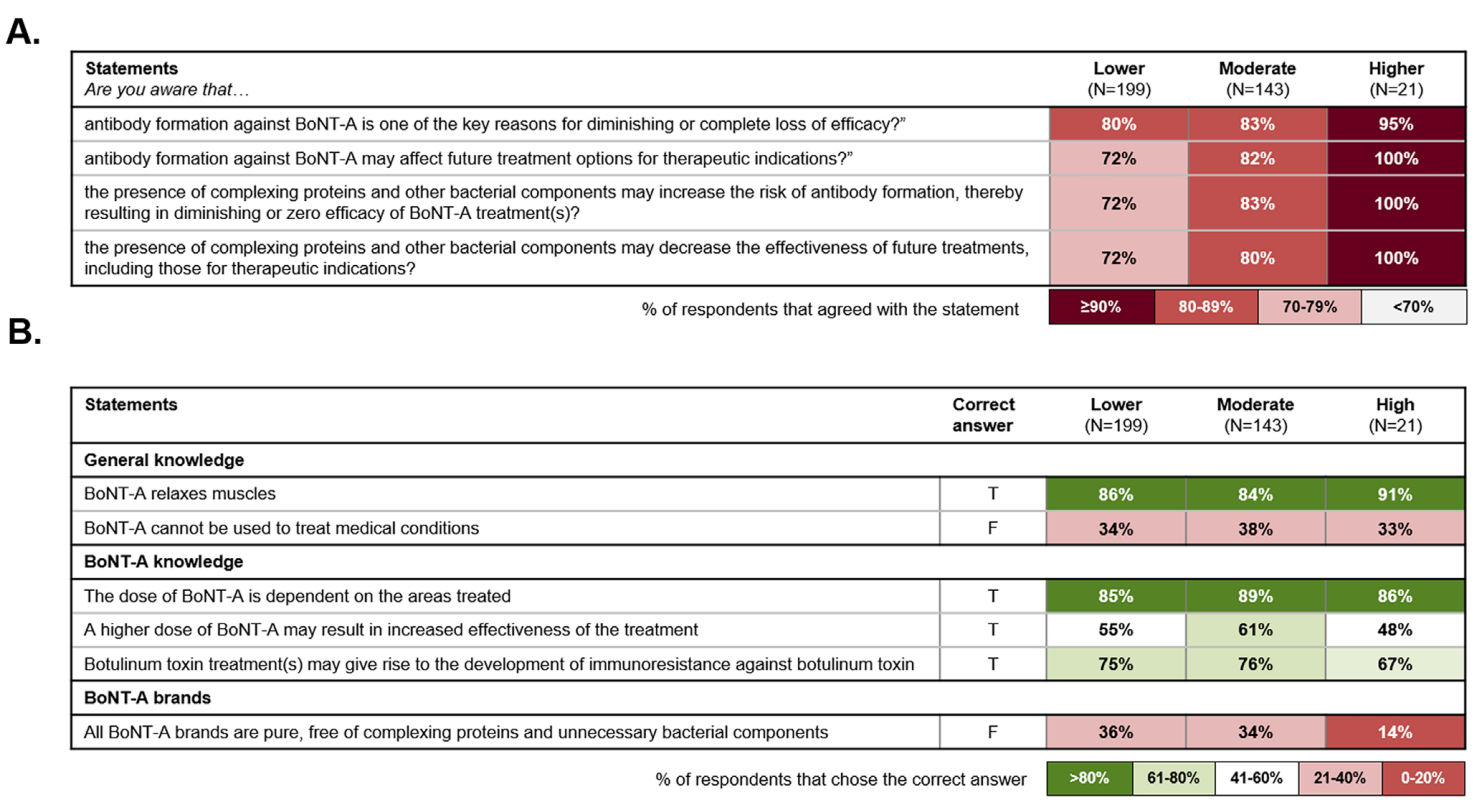
Differences between latent risk profiles in terms of (A) self‐reported awareness and (B) knowledge and understanding of BoNT‐A immunoresistance.

The identified risk profiles did not differ notably in terms of their sociodemographic variables (data not shown). However, there were notable differences in terms of participants' self‐reported awareness, knowledge, and attitudes toward BoNT‐A treatment and associated immunoresistance risk. The “higher‐risk” group claimed greater awareness of the potential negative impact of NAb formation and complexing proteins on BoNT‐A treatment efficacy. All “higher‐risk” respondents reported they were aware of how NAb formation could affect future treatment options and understood the role of complexing proteins and bacterial components in increasing immunoresistance risk. 95% acknowledged the role of NAb formation in diminishing BoNT‐A treatment efficacy, a higher percentage than observed in the “moderate‐risk” and “lower‐risk” profiles (Figure 4A).

Despite reporting greater awareness of potential risks, the “higher‐risk” group demonstrated poorer knowledge of how BoNT‐A treatments work and the differences among formulations compared with the “lower‐risk” group. While 91% of those in the “higher‐risk” group correctly recognized that BoNT‐A relaxes muscles, only 33% accurately identified that BoNT‐A can be used to treat medical conditions, similar to the “lower‐risk” group (34%). Knowledge gaps regarding BoNT‐A brands were especially evident among the “higher‐risk” group, only 14% of whom correctly recognized that not all brands are free of complexing proteins and unnecessary bacterial components; in contrast, 36% of participants in the “lower‐risk” group correctly answered this question (Figure 4B). These data suggest that the “higher‐risk” group have poor understanding of BoNT‐A and immunoresistance, but are not aware of their knowledge gaps.

**FIGURE 5:**
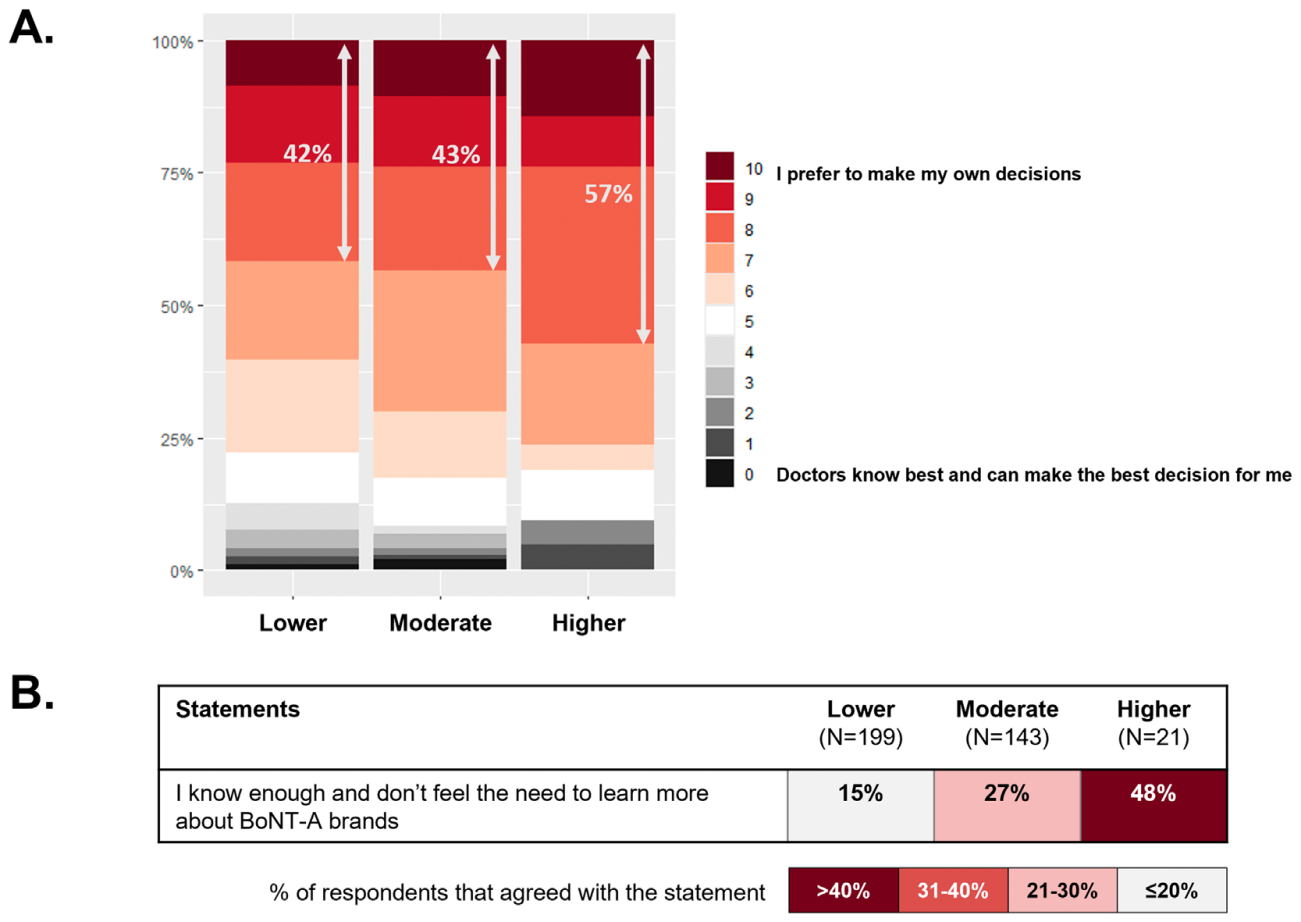
Differences between latent risk profiles in terms of attitudes toward BoNT‐A treatment‐related information and decision‐making.

Individuals within the “higher‐risk” profile exhibited a stronger preference for autonomy in their treatment decisions (57%), in contrast with the “moderate‐risk” (43%) and “lower‐risk” (42%) groups who rely more heavily on their doctors in the decision‐making process (Figure 5A). They also demonstrated notable confidence in their understanding of BoNT‐A immunoresistance, believing themselves to be sufficiently informed and seeing no need for additional education (Figure 5B). This contrasts with their poor knowledge and understanding of BoNT‐A immunoresistance (Figure 4B), highlighting their lack of insight.

**FIGURE 6:**
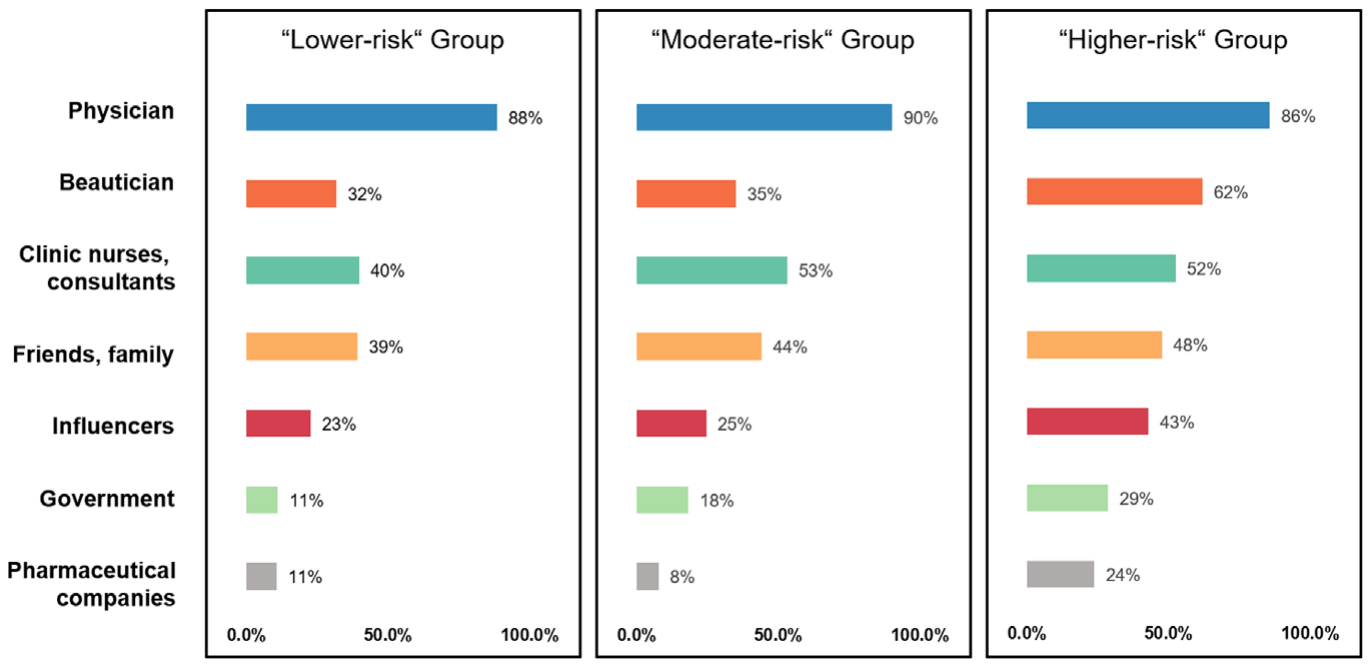
Differences between latent risk profiles in terms of sources of advice on BoNT‐A treatment.

The analysis also revealed differences in information‐seeking behaviors among risk groups. Whereas the majority of the respondents indicated relying on physicians (dermatologists, plastic surgeons, aesthetic physicians, and general practitioners) for advice on BoNT‐A treatment and product selection, the “higher‐risk” group stands out for its greater likelihood to also consult beauticians in beauty salons with 62% of this group seeking such advice, noticeably higher than the “lower‐risk” (32%) and “moderate‐risk” (35%) groups. This trend extends to other nonphysician sources of information as well (Figure 6). This pattern suggests a broader and more diverse approach to gathering information on BoNT‐A treatments among those in the “higher‐risk” group.

## 4 Discussion

Within this sample of experienced aesthetic BoNT‐A recipients, we identified three distinct profiles (“lower‐risk”, “moderate‐risk”, and “higher‐risk”) based on LCA and the five defined risk proxy variables. The results are consistent with a continuum from higher to lower risk with multiple contributing factors. The clear differentiation among risk profiles based on proxies of BoNT‐A exposure (i.e., the number of treatments received and areas of the face/body treated) was consistent with the observed trend in the number of symptoms suggestive of declining BoNT‐A efficacy. This concurrence with known risk factors (BoNT‐A dose, number, or interval of treatments) supports the validity of this proxy‐based approach for analyzing potential risks of immunoresistance. Additionally, our observations suggest that a structured or visual way of eliciting BoNT‐A treatment history information (number of treatments, areas treated, similar to the treatment area score used in our analysis) may be useful during in‐clinic consultations to facilitate discussions on immunoresistance risk with patients. Such scoring systems can also be used in public educational campaigns to increase awareness of BoNT‐A immunoresistance risk among patients.

Next, we explored how behavioral factors, in this case, switching between aesthetic clinics or providers and BoNT‐A formulations, were related to the risk profiles. There was a clear differentiation between the lower and higher ends of the risk continuum in terms of clinic switching behavior, with the “lower‐risk” group being most clearly differentiated from the other two. It has been suggested that an individual's decision to switch clinics/practitioners could be motivated by dissatisfaction with current treatment providers due to a perceived decline in BoNT‐A treatment efficacy over time, which could in fact be a sign of developing immunoresistance. Driven by the desire to achieve better aesthetic outcomes, this clinic switching behavior poses a practical challenge to practitioners in documenting their patients' full BoNT‐A treatment history and may result in patients receiving additional or more frequent injections, which ultimately contributes to increased BoNT‐A exposure. The reported frequency of brand switching showed less clear differentiation among the profiles. This is not unexpected since some reasons given by participants for switching BoNT‐A formulations were potentially immunoresistance‐related (diminished treatment effects, needing more frequent or higher dose treatments), whereas others appeared unrelated (cost or recommendations from others). Overall, these results indicate that, although brand and clinic switching behaviors may be associated with immunoresistance risk, the underlying relationships are complex and require further research for a comprehensive understanding.

Our results revealed other, less obvious, participant characteristics that may influence their treatment‐seeking behavior. For example, although the “higher‐risk” group claimed a high awareness of the risks associated with NAb formation, their overall understanding of BoNT‐A treatment and the distinctions between different formulations was less comprehensive than that of their “lower‐risk” counterparts. Moreover, they also placed a high value on autonomy and expressed confidence in their understanding of treatment implications. It is possible that the individuals in the “higher‐risk” group may be experiencing the Dunning–Kruger effect [14, 15], where overestimation of their understanding may influence their treatment‐seeking behaviors in ways that inadvertently elevate their risk. Interestingly, individuals in the “higher‐risk” group also exhibited a distinct pattern of information‐seeking behavior. They were more likely to seek advice and information from diverse sources, including beauticians and influencers, besides professional medical or governmental sources. Reliance on potentially less medically rigorous information could contribute to the observed knowledge gaps. Moreover, price sensitivity is another factor influencing treatment‐seeking behaviors that could lead to elevated risk (e.g., choosing cheaper BoNT‐A brands that may not have high purity and/or low immunogenicity). This suggests a potential area for targeted patient engagement and public education initiatives.

Understanding the complex behavioral factors and biological factors that contribute to BoNT‐A immunoresistance risk could guide interventions that positively influence behaviors to help individuals mitigate risk. Our observations provide new insights into how aesthetic patients' knowledge and attitudes may influence their treatment‐related behaviors, and potentially their risk of developing BoNT‐A immunoresistance. With this understanding, practitioners can increase engagement in shared decision‐making, which can lead to better‐informed choices and long‐term treatment satisfaction and safety. During consultations with individuals seeking their first BoNT‐A treatment, HCPs should ideally provide basic information on how BoNT‐A treatments work, the importance of starting treatment with a formulation of high purity and low immunogenicity, and the rationale for their recommended treatment intervals and doses. For experienced BoNT‐A recipients, consultations should ideally include a review of treatment history and actively solicit information on concurrent aesthetic or medical BoNT‐A treatments, since these contribute to increased total BoNT‐A exposure. Although our dataset did not contain data on concurrent medical/aesthetic treatments, the “crossover use” of BoNT‐A across different disciplines may become a more prominent issue as aesthetic BoNT‐A use continues to grow. Lastly, the results suggest that it is important to be attentive to signs of declining efficacy, whether from patient reports or clinical observations of dose and interval “creep” [4] and to take steps to assess and manage SNR in a timely manner.

A strength of our study is the novel application of a person‐oriented approach to explore the issue of BoNT‐A immunoresistance risk. Our findings provide insights on informative risk proxies, and on how potential risk for individuals may vary within a population. These observations warrant further exploration and validation across different samples. Certain limitations of this study should be noted. First, the risk proxies used are indirect indicators, not direct measurements of clinical outcomes or NAb titers. However, the patterns observed for proxies of BoNT‐A exposure (e.g., treated areas) are consistent with what is expected for known risk factors from clinical studies, providing a measure of validation for our analytical approach. Second, although the dataset we used was generated for a different purpose (studying BoNT‐A treatment experiences, attitudes and understanding of immunoresistance), the sample was relevant for our intended analysis: individuals who had received multiple (≥ 6) BoNT‐A treatments in the past 3 years and planned to continue doing so. Lastly, the study's modest sample size, particularly the small size of the “higher‐risk” group, limits our ability to draw general conclusions about the size of the effects observed. The present work could potentially be extended through purpose‐built surveys that capture additional risk proxies (e.g., improved identification of doses by indication, formulations, or concurrent treatments) and that include larger samples to increase the robustness of conclusions drawn.

## 5 Conclusion

Our analysis shows that consumer behaviors appear to influence the risk of BoNT‐A immunoresistance, but it is not straightforward to elucidate the underlying interrelationships with “biological” risk factors. Although challenging, a better understanding of the “human factor” may facilitate more targeted and effective interventions and shared decision‐making to mitigate risk.

## Author Contributions

All authors participated in conceptualization and design of the analysis, data interpretation, writing, reviewing, and editing of the manuscript. All authors have read and approved the final manuscript.

## Ethics Statement

The authors confirm that the ethical policies of the journal, as noted on the journal's author guidelines page, have been adhered to. No ethical approval was warranted for this secondary analysis of an anonymized consumer survey dataset previously published (ref. [5]; Corduff et al., 2024).

## Conflicts of Interest

Dr. Park serves as a consultant, trainer, and speaker for Merz Aesthetics. Dr. Tseng serve(d) as a speaker, trainer, and advisory board member for Merz Aesthetics, AbbVie, Bausch & Lomb, Cynosure, Galderma, and Solta. Dr. Vachiramon serves as a speaker for Merz Aesthetics, LG Chem, Leo Pharma, Beiersdorf, L'Oreal, and as an advisory board member for Merz Aesthetics, AbbVie, and L'Oreal. Dr. Gold serves as a consultant for Merz Aesthetics and conducts clinical studies for Merz Aesthetics. He serves as editor‐in‐chief of the Journal of Cosmetic Dermatology. Dr. Pavicic serves as a consultant and speaker for Merz Aesthetics and as an investigator for Merz Aesthetics, LG, AAT, and AbbVie. Dr. Tay is an employee of Merz Asia Pacific. G Toh is an employee of Tech Observer Asia Pacific. D Tan was an employee of Vista Health at the time that the research was performed.

## Supporting information


Table S1

## Data Availability

The datasets analyzed in this article are proprietary and not publicly available. Questions about the datasets should be directed to clifton.tay@merz.sg.
