# Supplementary figures and images for: Identifying Different Immunoresistance Risk Profiles Among Experienced Aesthetic Botulinum Neurotoxin A Recipients: A Latent Class Analysis
Source: J Cosmet Dermatol. 2024 Dec 8;24(2):e16686. doi: 10.1111/jocd.16686 (PMC11845912; doi:10.1111/jocd.16686)

# Tables

**Supplementary Table 1.** Evaluation of LCA model fit parameters for 2-, 3- and 4-class models

*
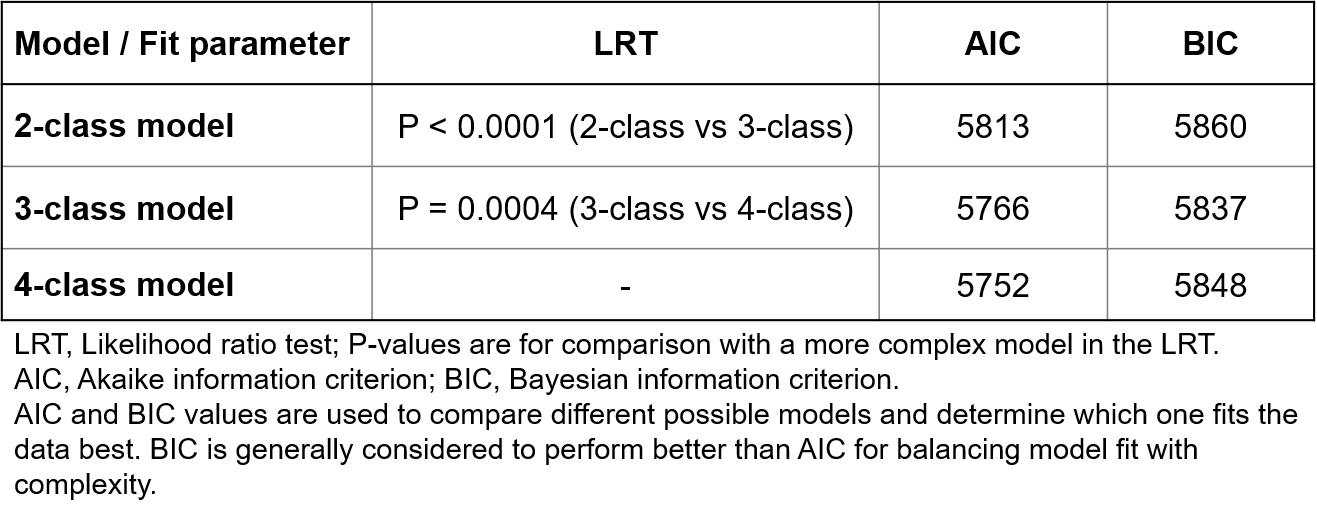
*

Supplement: Supplementary file 1 — Table S1 [file JOCD-24-e16686-s001.docx]
